# Prioritizing disease candidate genes by a gene interconnectedness-based approach

**DOI:** 10.1186/1471-2164-12-S3-S25

**Published:** 2011-11-30

**Authors:** Chia-Lang Hsu, Yen-Hua Huang, Chien-Ting Hsu, Ueng-Cheng Yang

**Affiliations:** 1 Institute of Biomedical Informatics, National Yang-Ming University, Taipei City, Taiwan 11221, Republic of China; 2 Department of Biochemistry, Faculty of Medicine, School of Medicine, National Yang-Ming University, Taipei City, Taiwan 11221, Republic of China; 3 Center for Systems and Synthetic Biology, National Yang-Ming University, Taipei City, Taiwan 11221, Republic of China

## Abstract

**Background:**

Genome-wide disease-gene finding approaches may sometimes provide us with a long list of candidate genes. Since using pure experimental approaches to verify all candidates could be expensive, a number of network-based methods have been developed to prioritize candidates. Such tools usually have a set of parameters pre-trained using available network data. This means that re-training network-based tools may be required when existing biological networks are updated or when networks from different sources are to be tried.

**Results:**

We developed a parameter-free method, interconnectedness (ICN), to rank candidate genes by assessing the closeness of them to known disease genes in a network. ICN was tested using 1,993 known disease-gene associations and achieved a success rate of ~44% using a protein-protein interaction network under a test scenario of simulated linkage analysis. This performance is comparable with those of other well-known methods and ICN outperforms other methods when a candidate disease gene is not directly linked to known disease genes in a network. Interestingly, we show that a combined scoring strategy could enable ICN to achieve an even better performance (~50%) than other methods used alone.

**Conclusions:**

ICN, a user-friendly method, can well complement other network-based methods in the context of prioritizing candidate disease genes.

## Background

The wide applications of high-throughput techniques have enabled researchers to investigate disease mechanisms in a genome-wide scale [1,2]. However, one challenge is that these techniques are usually unable to precisely pinpoint the causative genes. For example, a linkage analysis may give a disease-linked chromosomal region, which may harbor hundreds of candidate genes [3,4]; an association study may identify a number of false positives if the disease under investigation has a complex inheritance pattern [5]. While a whole genome re-sequencing can find a number of genetic variations in a patient, only a few of them may play a role in the disease etiology [1]. Therefore, time-consuming and laborious experiments are usually required to determine the real disease genes from a large number of candidates given by high-throughput experiments. One strategy to accelerate the whole disease gene finding process is to use a computational approach to prioritize candidate genes.

Many computational approaches for prioritizing candidate genes have been developed, assuming that one disease could be caused by a group of functionally related genes. Such approaches measure the functional similarity of each candidate gene to known disease genes using experimentally verified biological data (for details see review [6-9] and Additional File 1). Among these approaches, network-based ones have shown a good performance. The working hypothesis of network-based methods is that genes causing one disease are likely to locate closely to each other in a biological network [6,10]. Some network-based methods prioritize candidate genes based on whether they directly interact with known disease genes [11,12]; other methods further consider the shortest-path distance between candidate genes and known disease genes in a network when direct links do not exist [13,14]. On the other hand, different methods might employ distinct scoring strategies. Lage *et al. *[15] developed a Bayesian predictor that could combine interactome and phenome to infer putative protein complexes likely to associate with a disease. The CIPHER method scores the candidate genes using a regression model of phenotype similarity and gene closeness in a network [16]. Other network-based algorithms, such as random walk [17], network flow [18], page rank [19], network partition [20], and network clustering [21], were also designed to prioritize candidate disease genes.

**Figure 1:**
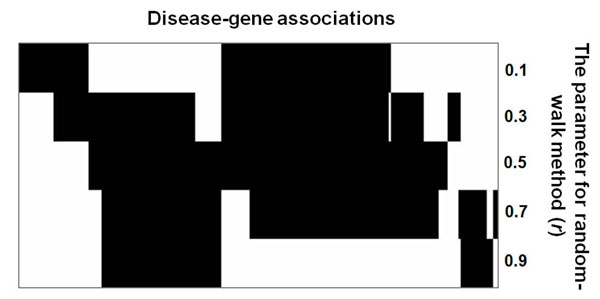
**Associations between parameter values and disease-gene association predictions**. There are 220 disease-gene association cases in this example. The parameter *r* is used in random walk method to control the probability of returning to initial node [17]. The solid blocks indicate this method with a specific parameter value successfully gives the true disease genes the highest ranking (for details see the Materials and methods).

Network-based methods usually have some parameters that need to be trained using available data sets. The random walk method needs a parameter to control the probability of returning to the initial node [17], and the network flow algorithm uses a parameter to describe the relative importance of prior information [18]. Lage’s method requires determining several parameters in order to build the predictor [15]. Whenever biological networks are updated or new training data become available, their parameters should be re-tuned in order to optimize their performance. It may be difficult for biologists to rep eat these processes by themselves. Additionally, a parameter set may just work for certain cases. Here, we take the random walk (RW) method as an example. Although a parameter setting (*r* = 0.5) of RW appears to suffice the identification of many disease genes, using other parameters may be required to find certain disease genes (Figure 1). How to intelligently choose the parameters could be a difficult task to users. We argue that a parameter-free algorithm could be more useful to users in this regard.

In this study, we propose a new candidate gene prioritization approach that measures the interconnectedness (ICN) between genes in a network. It was designed to be a parameter-free method. Unlike other network-based methods, ICN measures closeness of each candidate genes to known disease genes by taking alternative paths into consideration, in addition to the direct link and the shortest-path distance. In comparison with other outperforming network-based methods, ICN is a competitive method. In particular, we show that an impressive performance of prioritizing candidate disease genes could be achieved by combining ICN with other network-based methods. Finally, a novel type of spinocerebellar ataxia (SCA) was chosen to demonstrate the ability of this method.

## Results and discussion

### Principles of the interconnectedness-based method

Most network-based gene prioritization methods, including this one we have developed here, were created on the basis that causative genes of one disease may tend to locate closely in the network [6,10]. The approaches taken by various methods differ on how closeness between genes is measured. Before this method is developed, other network-based methods prioritize candidate genes by finding direct-linked disease genes or close disease genes using shortest-path distance. One concern with these previous methods is that they might be less effective than expected if there are noises or missing direct links in the network used to measure inter-gene closeness. Consequently, we designed the InterConnectedNess-based method, ICN, to measure the closeness between genes by considering alternative paths, in addition to the shortest one, that could connect candidate genes to known disease genes. Briefly, ICN determines that these genes are more likely to belong to the same functional module if two genes have more shared interacting genes. A functional module may correspond to a protein complex [15,18] or to a signalling pathway [22]. If a functional module is implicated with a disease, changes to a member gene in this module may cause this disease [23,24]. We applied ICN to the problem of prioritizing disease candidate genes.

### Comparison with other network-based prioritization algorithms

According to the comprehensive comparison performed in [25], the best two outperforming methods for prioritizing candidate genes were the Random Walk method (RW) [17] and the PRINCE (PRIoritizatioN and Complex Elucidation) algorithm (PR) [18]. In this project, they were re-implemented in order to compare their performance with that of ICN. Their parameters were optimized as described in [18] (for details see Materials and Methods).

Two biological networks were recruited as the data sets to evaluate the performance of ICN and other two methods. These networks were chosen because each network has features distinctive from that of the other. We intended to examine if each method could perform in a consistent manner using different types of network data. The first one is a protein-protein interaction network (PIN) consisting of 140,382 interactions and 12,164 genes. PIN consists of data retrieved from nine protein-protein interaction data sources [26-34]. The second one is a functional association network (FAN) consisting of 1,217,908 interactions and 16,648 genes downloaded from the STRING database [35]. These two networks share 11,776 common genes and 95,630 common interactions. Two major differences between these data sets are the number of interactions and the types of edges. While PIN edges are un-weighted, FAN edges are annotated with weights indicating the confidence of functional linkage between each pair of connected genes [36]. ICN is able to incorporate edge weights in quantifying the closeness between genes in a network. The statistics of available data in each network is summarized in Table 1.

**Table 1:** Statistics of biological networks

	Protein-protein interaction network (PIN)	Functional association network (FAN)
Data source(s)	Integration from DIP, BOND, IntAct, MINT, MIPS, HPRD, BioGRID, Reactome, and pathway commons	STRING v8.2
Network type	Unweighted	Weighted
# genes	12,164	16,648
# interactions	140,382	1,217,908
# disease families	344	509
# disease-gene associations	1,993	2,616
# disease genes	1,640	1,909

A leave-one-out procedure was employed to carry out the evaluation. The disease-gene associations were obtained from OMIM [37]. These genes were manually grouped in to different disease families as described in Materials and Methods. In each validation trial, the association of one test gene with a disease family was removed, and each method was tried to re-build this association. To mimic the situations we may encounter when using different high-throughput genome-wide techniques to find disease genes, we created two test scenarios, the simulated linkage analysis and the whole genome scan. In the simulated linkage analysis, each time a test disease gene together with 100 genes on its flanking regions was taken as the candidate set. In the whole genome scan, each time a test disease gene together with all human genes in the network, excluding other members from the corresponding disease family, was taken as the candidate set. If a test gene was ranked top *k* in a candidate set in a trial, this trial was regarded as a successful one. We further defined the “success rate” as the fraction of successful trials for a method under a particular test scenario.

**Figure 2:**
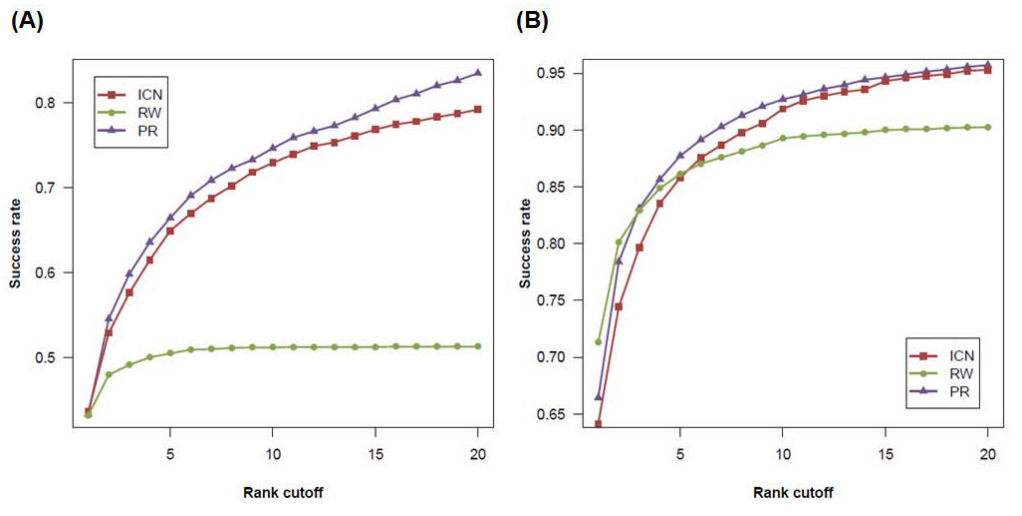
**A performance of prioritization methods tested on simulated linkage analyses**. The performance of different methods is assessed by using PIN (A) and FAN (B), respectively.

The results of simulated linkage analysis for each method are presented in Figure 2. 1,993 and 2,616 disease-gene associations were tested using PIN and FAN, respectively. When PIN was used, ICN achieved the best performance with a success rate of 44.7%, ranking the known disease genes as top 1 candidate (*k*=1) in 870 out of 1,993 cases. RW and PR also achieved the similar performance with a success rate of 43.3% (862/1993) and 43.4% (865/1993), respectively. When the rank cutoff (*k*) was increased, PR had the best performance, while the performance of ICN was still comparable with that of PR (Figure 2A). When FAN was used, RW achieved a success rate of 71.3% (1865/2616), better than that ICN (64.1%, 1678/2616) and PR (66.4%, 1738/2616) did. On the other hand, as rank cutoff was increased (*k* >= 5), the performance of ICN and PR was better than that of RW (Figure 2B).

**Figure 3:**
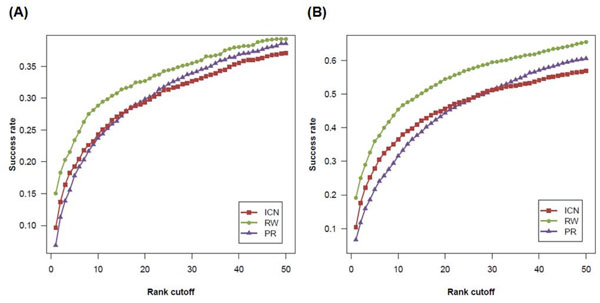
**A performance of prioritization methods tested on whole genome scans**. The performance of different methods is assessed by using PIN (A) and FAN (B), respectively.

The performance comparison under the test scenario of whole genome scan is shown in Figure 3. When PIN was used, ICN successfully ranked the known disease genes as top 1 candidate in 192 out of 1,993 cases, with a success rate of 0.096. RW performance with a success rate of 15.0% (299/1,993) was higher than ICN and PR (6.9%, 137/1,993). Similarly, the performance of ICN (10.4%, 272/2,616) was between RW (19.1%, 499/2,616) and PR (6.7%, 174/2,616) when FAN was used. The benchmark reveals that although ICN did not outperform in all cases, it was quite comparable to other methods.

**Figure 4:**
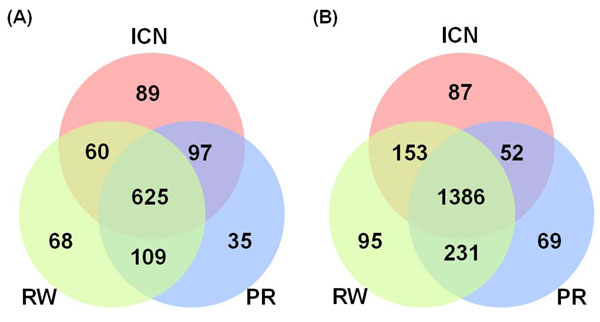
**Venn diagram of successfully predicted cases among different prioritization methods.** The cases which are successfully ranked the known disease genes as top 1 candidate are compared among ICN, RW, and PR by using PIN (A) and FAN (B), respectively.

If the cases with disease genes being ranked as top 1 candidates by at least one of three prioritization methods were considered as successful predictions, the overall success rates so achieved were 54.3 % (1,083/1,993) by using PIN and 79.2% (2,073/2,616) by using FAN, respectively, under the test scenario of simulated linkage analysis. The overall performance was much better than that of respective methods. Figure 4 presents the overlaps of successful predictions among ICN, RW, and PR. No matter which biological network was used, RW and PR shared more success cases than other combinations. This is not really surprising, since RW and PR took a similar iterative procedure to look for candidate genes in a network [17,18]. Interestingly, each method predicted unique cases. In particular, ICN gave the highest number of unique success cases using PIN, and it gave a comparable number of unique cases with that of RW using FAN. These results indicate that each method may perform better than other methods on certain cases. Analyzing the difference of the unique success cases generated by different methods may help us get a deeper understanding of unique advantage of each method, which could assist us to further improve the performance.

### Exploring the cases uniquely predicted by respective methods

Intuitively, topological properties of genes in a network may affect the performance of candidate gene prioritization when network-based methods are used. To understand how the performance of different methods could be influenced, we examined if the disease genes uniquely identified by individual methods had distinctive topological properties. For simplicity, disease genes uniquely identified by ICN are denoted as ICN-unique genes/cases, and so forth for other methods, in the following text.

**Figure 5:**
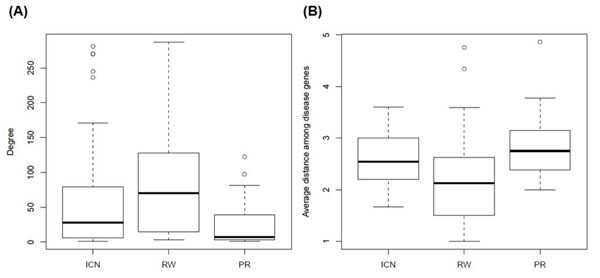
**Analysis of network topological properties on disease causing genes.** The topological properties of disease genes in unique cases which were successfully ranked the known disease genes as top 1 candidate by a specific method in PIN (Figure 3A) were compared in degree (A) and average shortest-path distance between other disease-associated genes which are in the same disease family(B).

Firstly, the number of interacting partners, also referred to as the degree in the graph theory [38], of each method-unique case was considered. We noticed that when PIN was used, the average degree of RW-unique cases was significantly higher than these of ICN- and PR-unique cases (P-value = 0.002 and 2.9×10-6, Wilcoxon signed-rank test). Secondly, we explored to which extent a method-unique gene may be located, in a network, away from the known genes implicated in a disease family. Here we found that when PIN was used, the distribution of the shortest-path distances of ICN-unique cases is similar to that of PR-unique cases (Figure 5B). Both ICN-unique and PR-unique cases are significantly more distant from known disease genes than that of RW-unique cases (P-values = 1.9×10-5 and 2.6×10-5, respectively, Wilcoxon signed-rank test). The analysis of the method-unique cases using FAN yielded a similar result (Additional File 2).

On the whole, these results support that a prioritization method may outperform the others when candidate disease genes to be assessed have certain method-favored topological properties. When candidate genes have more interacting partners in a network and are closer to other known disease genes, RW may perform better than the other methods. In contrast, ICN and PR may outperform RW when prioritizing candidate genes that are more distant away from other known disease genes in a network. Therefore, it is quite possible that combining the ranking results of different methods may further improve the performance of candidate gene prioritization. In the next section, we show that a combined scoring strategy did improve the performance of prioritizing candidate disease genes.

### Improving the performance using a combined scoring strategy

Since each method may have its own favorite cases, we tried to improve the performance of prioritization by combining the results generated by different methods.

To preserve the unique advantage of each method, we did not change any algorithmic approaches in them. Instead, we used a combined scoring strategy by multiplying together the ranks generated by different methods (for details see section Materials and Methods). The performance of this new approach was also evaluated using the leave-one-out procedure under a test scenario of either simulated linkage analysis and whole-genome scan.

Table 2 lists the performances of respective methods and different combined scoring schemes tested in the simulated linkage scenario. Here, we denote the scoring scheme of combining the ranking results of ICN and PR as the ICN-PR method, and so forth. Interestingly, all combined scoring schemes achieved higher success rates than respective methods. When PIN was used, the ICN-PR method showed the best performance (success rate 48.9%). Besides, the ICN-RW method also showed a better success rate (46.9%) than respective methods. On the other hand, when FAN was used, the RW-PR method outperformed the other individual and combined methods (success rate 73.7%). The ICN-PR method achieved a success rate (72. 7%) close to the best one. All the combined scoring schemes made substantial performance improvement compared to respective methods (ICN: 64.1%, RW: 71.3%, PR: 66.4%). Finally, when these combined scoring schemes were tested in the whole genome scan scenario, no performance improvement could be found (data not shown). It is not surprising since we expect that there could be missing parts in currently available biological networks and more genes are yet to be identified to fill in the networks.

**Table 2:** Success rates of ranking known disease genes as the best candidate

Success rate (%)	ICN	RW	PR	ICN-RW	ICN-PR	RW-PR	ICN-RW-PR
PIN	43.7	43.3	43.4	46.9	48.9	46.8	44.5
FAN	64.1	71.3	66.4	72.7	73.3	73.7	72.4

Here we further explored if the cases failed when respective methods were used could be recovered using the combined scoring schemes. The result is listed in Table 3. When PIN was used, 11 and 25 cases (out of 911 cases failed using respective methods) could be recovered by the ICN-RW and the ICN-PR methods, respectively, but no cases could be recovered by the RW-PR method or the ICN-RW-PR method. We also tested if it could make a difference if FAN was used. It turned out that the ICN-RW method and the ICN-PR method rescued 27 and 22 cases (out of 543 cases failed using respective methods), respectively. The RW-PR method could rescue only one case, and the ICN-RW -PR method did not really show a much better performance (4 cases rescued).

**Table 3:** Failed prediction cases recovered by combined methods

	# failed prediction cases^&^	# cases re-ranked as top 1 candidate			
	
		ICN-RW	ICN-PR	RW-PR	ICN-RW-PR
PIN	911	11	25	0	0
FAN	543	27	22	1	4

^&^ a failed prediction case indicates that no prioritization method can rank the true disease gene as top 1 candidate.

All in all, combining the results of different network-based methods indeed enhances the performance of prioritizing candidate disease genes. In particular, substantial performance improvement was made when combining ICN with other methods.

### Using ICN and combined scoring schemes to find spinocerebellar ataxia genes

To demonstrate the ability of ICN and the combined scoring schemes in finding novel disease genes, we present a case study for spinocerebellar ataxia type 22 (SCA22) [39]. Autosomal dominant spinocerebellar ataxias (SCAs) are a group of progressive neurodegenerative disorders characterized by the loss of balance and motor coordination due to dysfunction of the cerebellum [40]. SCAs are genetically heterogeneous. To date, more than 30 genomic loci have been linked to different subtypes of SCA; however, only 18 causative genes have been determined [41,42]. Interestingly, these genes share common interacting partners [43], suggesting that network-based methods could be suitable for finding novel SCA-causing genes. SCA22 has been found to link to the locus on chromosome 1q21-23 [39], where 541 protein-coding genes were annotated (Ensembl release 58, http://www.ensembl.org). Our aim was to prioritize these 541 candidate disease genes.

The confirmed SCA-causing genes in Table 4 were regarded as known disease genes for the SCA disease family. There were 15 and 17 of them in PIN and FAN, respectively. Table 5 and 6 present the top 10 candidate genes (*i.e. k* = 10) prioritized using PIN and FAN, respectively. Firstly, we tested individual methods. We noticed that ICN, RW, and PR generated very different results. No identical top one gene could be consistently determined by different methods. In addition, when PIN was used, only 2 genes, SPTA1 and GNAT2, were commonly identified by all methods (*k* = 10, Table 5). Similarly when FAN was used, only 3 genes (KCNN3, SPTA1, and KCNC4) commonly identified by all methods (*k* = 10, Table 6).

Secondly, we tested combined scoring schemes and they appeared to generate more consistent results. When PIN and FAN were used respectively, there were correspondingly three (SPTA1, GNAT2, and NRAS) and seven (KCNN3, SPTA1, CCT3, KCNC4, KCNA2, KCND3, and KCNA3) common genes identified by all combined scoring schemes (*k* = 10, Table 5 and 6). Furthermore, SPTA1 and KCNN3 were consistently picked out as the best candidates by all combined scoring schemes using PIN and FAN, respectively. SPTA1 was also ranked in the top 3 candidate genes by combined scoring schemes when FAN was used. KCNN3 was not included in the candidate list when PIN was used because there was no interaction information for KCNN3.

**Table 4:** List of SCA-causing genes

SCA subtype	Gene	PIN^&^	FAN^&^
SCA1	ATXN1	Y	Y
SCA2	ATXN2	Y	Y
SCA3	ATXN3	Y	Y
SCA5	SPTBN2	Y	Y
SCA6	CACNA1A	Y	Y
SCA7	ATXN7	Y	Y
SCA8	ATXN8	N	N
SCA10	ATXN10	Y	Y
SCA11	TTBK2	Y	Y
SCA12	PPP2R2B	Y	Y
SCA13	KCNC3	N	Y
SCA14	PRKCG	Y	Y
SCA15	ITPR1	Y	Y
SCA17	TBP	Y	Y
SCA27	FGF14	N	Y
SCA28	AFG3L2	Y	Y
SCA31	PLEKHG4	Y	Y
DRPLA	ATN1	Y	Y

^&^ whether the disease genes are in the given network. DRPLA: dentatorubral-pallidoluysian atrophy

**Table 5:** Top 10 candidate genes for SCA22 by using PIN

Rank	ICN	RW	PR	ICN-RW	ICN-PR	RW-PR	ICN-RW-PR
1	SPTA1	NRAS	YY1AP1	SPTA1	SPTA1	SPTA1	SPTA1
2	GNAT2	SPTA1	ECM1	GNAT2	YY1AP1	AHCYL1	GNAT2
3	TAF13	GNAT2	AHCYL1	NRAS	GNAT2	NRAS	NRAS
4	ISG20L2	GNAI3	FDPS	ISG20L2	TAF13	ECM1	YY1AP1
5	FCGR2C	AHCY1	SPTA1	FCGR2C	ECM1	YY1AP1	ECM1
6	YY1AP1	STXBP3	STXBP3	TAF13	NRAS	GNAT2	TAF13
7	PSMD4	ECM1	S100A7	PSMD4	STXBP3	STXBP3	STXBP3
8	NRAS	CCT3	POLR3C	GNAI3	PSMD4	GNAI3	AHCYL1
9	NGF	RPS27	UBAP2L	NGF	POLR3C	S100A7	GNAI3
10	NTRK1	S100A7	GNAT2	NTRK1	NGF	FDPS	PSMD4

**Table 6:** Top 10 candidate genes for SCA22 by using FAN

Rank	ICN	RW	PR	ICN-RW	ICN-PR	RW-PR	ICN-RW-PR
1	S100A6	SPTA1	KCNN3	KCNN3	KCNN3	KCNN3	KCNN3
2	KCNN3	CCT3	HCN3	SPTA1	S100A6	SPTA1	SPTA1
3	NGF	KCNN3	SPTA1	S100A6	SPTA1	CCT3	S100A6
4	KCNA2	S100A11	PPM1J	KCNA2	KCNC4	HCN3	CCT3
5	KCNC4	KCNA2	RHBG	CCT3	PPM1J	KCNC4	KCNC4
6	KCND3	KCNA3	KCNC4	KCNC4	KCNA2	KCNA2	KCNA2
7	KCNA3	KCND3	AHCYL1	KCND3	KCND3	S100A11	KCND3
8	SPTA1	KCNC4	CCT3	KCNA3	CCT3	PPM1J	KCNA3
9	HIST2H2BE	ARHGEF11	PYGO2	NGF	KCNA3	KCNA3	PPM1J
10	SHC1	CD5L	F11R	F11R	F11R	KCND3	F11R

From protein function and literature survey, we found that SPTA1 and KCNN3 are very likely to associate with SCA22. SPTA1 is a member of spectrin family, functioning in actin crosslinking and as the molecular scaffold proteins to determine cell shapes and to arrange the transmembrane proteins. An in-frame deletion in SPTBN2, which is also a member of the spectrin family, can cause SCA5 [44]. Recent studies have shown that the mutant SPTBN2 disrupts fundamental intracellular transport processes in synapses [45-47]. This is likely to contribute to progressive neurodegenerative disease, such as SCA. Therefore, SPTA1 may cause SCA22 in a similar mechanism. Besides, KCNN3 is a member of the gene family encoding the small conductance calcium-activated potassium channels. A CAG repeat polymorphism has been annotated in the amino-terminal coding region of KCNN3 [48]. Many studies revealed that such repeat polymorphisms associate with psychiatric diseases, such as schizophrenia [49] and bipolar diseases [50].

To further validate these two candidates experimentally, an exome sequencing experiment was performed, and several novel gene variations have been found on SPTA1 in two SCA22 patients (Chung, M.-Y. *et al.*, unpublished data). This preliminary result we present here suggests that ICN and the combined scoring schemes are able to identify the novel disease genes.

## Conclusions

The InterConnectedNess-based method (ICN) is a biologically intuitive and parameter-free approach for prioritizing candidate disease genes. There is no need for users to train the parameters every time when biological networks to be used are updated. ICN not only was comparable to other well-known methods, such the random walk method (RW) and the PRINCE algorithm (PR), but also outperformed these methods when candidate disease genes are located more distantly to known disease genes in a network. Furthermore, combined ICN-RW or ICN-PR scoring schemes showed an impressive performance improvement in prioritizing candidate disease genes, suggesting that different network-based methods may complement the weakness of each other.

In this study, we created a very simple combined scoring strategy by multiplying the ranks generated by different methods. The success of this strategy implies that there might still be a chance to further improve the performance of network-based methods in prioritizing candidate disease genes. To achieve this, we plan to try other strategies. In addition to combining method-specific ranking results, combining network-specific ranking results appears to be another promising strategy. In fact, two algorithms, N-dimensional order statistics (NDOS) [51] and discounted rating system (DRS) [52], have been employed in some prioritization methods to combine ranking results generated respectively by using different network data sets. It would be interesting to find out if the performances of ICN or other network-based methods can still be advanced when more heterogeneous approaches are integrated together.

## Materials and methods

### Biological networks

Two kinds of biological networks were employed to test the performance of network-based methods in this study: protein-protein interaction network (PIN) and functional association network (FAN). PIN was constructed by integrating protein-protein interaction data from nine databases, including DIP [26], BIND [27], IntAct [28], MIPS [29], MINT [30], HPRD [31], BioGRID [32], Reactome [33], and Pathway Commons [34]. Another dataset, FAN, was obtained from STRING v8.2, which was a comprehensive gene association dataset containing directly physical interactions and functional links from experimental evidence and computational methods [35]. In both networks, the identifier for each gene was mapped to Entrez Gene ID, and self-interacting pairs were removed. Finally, PIN consists with 140,382 interactions and 12,164 genes, and FAN consists of 1,217,908 interactions and 16,648 genes (Table 1). Each connection in FAN was assigned a confidence scores from STRING, which reflects the confidence of each gene-gene association. PIN and FAN were regarded as unweighted and weighted networks, respectively.

### Disease-gene associations

The disease-gene associations were retrieved from the Morbid Map in OMIM [37]. If the causative genes were not included in the networks, their associations to diseases were removed. Because the prioritizing methods require related disease genes for prediction, the related causative genes were manually grouped into a disease family based on their given disorder name [53], and disease families that have only one causative gene were filtered out. In total, 1,993 disease-gene associations implicated with 344 disease families were recruited in PIN and 2,616 disease-gene associations implicated with 509 disease families were recruited in FAN (Table 1).

### Interconnectedness (ICN) between genes

**Figure 6:**
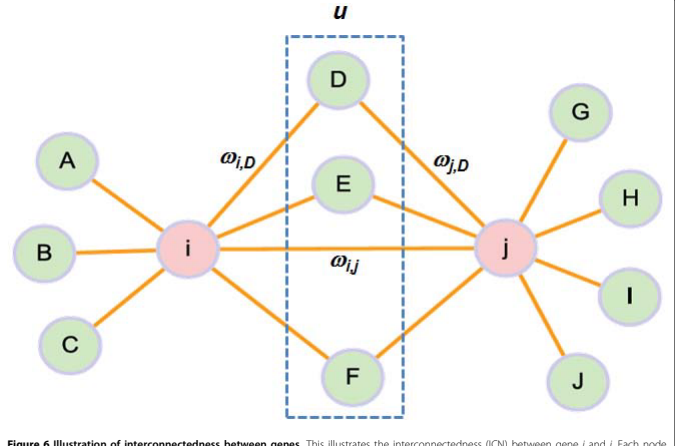
**Illustration of interconnectedness between genes.** This illustrates the interconnectedness (ICN) between gene *i* and *j*. Each node represents a gene and each edge represents a either physical interaction or functional association. *ω* is the weight of each connection. *u* is the set of connectors, which interact with both gene *i* and *j*.

The closeness between genes in a network was quantified by considering not only direct interaction of two genes but also the number of connectors between genes. As illustrated in Figure 6, the interconnectedness score *ICN_i_*_,_*_j_* between two genes *i* and *j* was defined as:(1)

where *N* is the neighboring genes of a given gene, and *u* is the gene linked to both gene *i* and *j*. *ω* is a weight of the connection between two genes, *e.g. ω_i_*_,_*_j_* corresponds to the weight between gene *i* and *j*. In FAN, the value of *ω* is within the interval between 0 and 1. In PIN, however, *ω* is either 1 or 0, *i.e.* connected or unconnected. Because the number of connectors may be associated with the number of neighbors of each node, the number of connectors between two genes is normalized by the expected number of connectors between these genes. *k_i_* is the sum of weights of gene *i*’s neighboring connections and is defined as:(2)

In an unweighted network, *k_i_* corresponds directly to the degree, namely the number of neighbors of a given gene [38].

### Prioritizing candidate genes by interconnectedness scores

Candidate genes are then prioritized based on the *ICN* scores calculated using equation 1. For a given disease *d*, each candidate gene was scored by summing up the closeness to the seed genes *S_d_*, *i.e.* the genes in the same disease family. The score of a given candidate gene *i* was calculated as:(3)

where *ICN_i_*_,_*_j_* is the connection score between gene *i* and *j*. All candidate genes are then ranked based on these scores.

### Implement of random walk (RW) and PRINCE (PR) methods

Both the random walk (RW) method [17] and the PRINE (PR) algorithm [18] apply an iterative procedure to find candidate disease genes in a network. When the difference between results of the previous and current steps (measured by *L*_1_-norm) fell below 10^-10^, the iteration was halted, and candidate genes were ranked based on the scores in the final step.

The precise behaviors employed by the two methods to reach candidate genes in a network differ. RW [17] simulates a random walker that starts from one or a set of source nodes, and moves forward to neighboring nodes with a probability proportional to the weight of the connecting edge. RW also allows the walker to move back to the source node with probability *r* in each step. *r* controls how far the random walker could get away from the source node. PR [18], a propagation-based algorithm, exploits prior information on causative genes for the same disease or similar ones and infers a strength-of-association function to smooth over the network (*i.e.* adjacent nodes are assigned similar values). The parameter *α in* PR controls the relative importance of prior information. Using the tuning procedure described in [18], we set *r* = 0.5 and *α* = 0.9, which make corresponding methods achieve the optimal performance when the two network data sets described in this study are used.

### Experiment design and performance measurement

Two test scenarios were designed to evaluate the performance of all methods: simulated linkage analyses and whole genome scan. In the simulated linkage analysis, a total of 100 genes flanking a test disease gene were taken as the candidate genes. In the whole genome scan, a test disease gene and all the genes in a biological network excluding other members from the corresponding disease gene family constitute the candidate gene list.

A leave-one-out procedure is used to assess the performance of the different methods. In each trial, a disease-gene association was removed and remaining genes in the same disease family were taken as seed genes to reconstruct the association. We used the “success rate” to represent the performance of a method. If the removed disease-gene association was ranked in top *k* of a candidate gene list, this trial was regarded as a successful prediction. The “success rate” of a method is defined as the fraction of successful predictions in all cases tested given a particular combination of a network data set and a test scenario.

### Combing the prioritization results given by different methods

For each candidate gene *i*, a combined score *CS_i_* was calculated as:(4)

where *R_i_*_,_*_j_* indicates the rank of gene *i* in method *j.* The candidate genes were re-ranked using the combined scores in an ascending order, *i.e.* the lower combined score, the higher priority.

## List of abbreviations used

FAN: functional association network; ICN: interconnectedness; OMIM: Online Mendelian Inheritance in Man; RW: random work method; PIN: protein-protein interaction network; PR: PRINCE algorithm.

## Authors' contributions

CLH conceived and designed the experiments. CLH and CTH performed the experiments. CLH, YHH and UCY analyzed and interpreted data. All the authors wrote and revised the manuscript.

## Competing interests

The authors declare that they have no competing interests.

## Supplementary Material

Additional file 1 List of related algorithms and tools for prioritizing disease candidate genes Click here for file

Additional file 2 **Analysis of network topological properties on disease causing genes** The topological properties of disease genes in unique cases which were successfully ranked the known disease genes as top 1 candidate by a specific method in FAN (Figure 3B) were compared in degree (A) and average shortest-path distance between other disease-associated genes which are in the same disease family(B). Click here for file

## References

[B1] Kuhlenbaumer G Hullmann J Appenzeller S Novel genomic techniques open new avenues in the analysis of monogenic disorders Hum Mutat 2011 32 2 144 151 10.1002/humu.21400 21280146

[B2] Tang WC Yap MK Yip SP A review of current approaches to identifying human genes involved in myopia Clin Exp Optom 2008 91 1 4 22 10.1111/j.1444-0938.2007.00181.x 18045248

[B3] Botstein D Risch N Discovering genotypes underlying human phenotypes: past successes for mendelian disease, future approaches for complex disease Nat Genet 2003 33 Suppl 228 237 12610532 10.1038/ng1090

[B4] Glazier AM Nadeau JH Aitman TJ Finding genes that underlie complex traits Science 2002 298 5602 2345 2349 10.1126/science.1076641 12493905

[B5] McCarthy MI Abecasis GR Cardon LR Goldstein DB Little J Ioannidis JP Hirschhorn JN Genome-wide association studies for complex traits: consensus, uncertainty and challenges Nat Rev Genet 2008 9 5 356 369 10.1038/nrg2344 18398418

[B6] Oti M Brunner HG The modular nature of genetic diseases Clin Genet 2007 71 1 1 11 17204041 10.1111/j.1399-0004.2006.00708.x

[B7] Zhu M Zhao S Candidate gene identification approach: progress and challenges Int J Biol Sci 2007 3 7 420 427 17998950 10.7150/ijbs.3.420 PMC2043166

[B8] Kann MG Advances in translational bioinformatics: computational approaches for the hunting of disease genes Brief Bioinform 2010 11 1 96 110 10.1093/bib/bbp048 20007728 PMC2810112

[B9] Tranchevent LC Capdevila FB Nitsch D De Moor B De Causmaecker P Moreau Y A guide to web tools to prioritize candidate genes Brief Bioinform 2011 12 1 22 32 10.1093/bib/bbq007 21278374

[B10] Ideker T Sharan R Protein networks in disease Genome Res 2008 18 4 644 652 10.1101/gr.071852.107 18381899 PMC3863981

[B11] Chen JY Shen C Sivachenko AY Mining Alzheimer disease relevant proteins from integrated protein interactome data Pac Symp Biocomput 2006 367 378 17094253

[B12] Oti M Snel B Huynen MA Brunner HG Predicting disease genes using protein-protein interactions J Med Genet 2006 43 8 691 698 10.1136/jmg.2006.041376 16611749 PMC2564594

[B13] Krauthammer M Kaufmann CA Gilliam TC Rzhetsky A Molecular triangulation: bridging linkage and molecular-network information for identifying candidate genes in Alzheimer's disease Proc Natl Acad Sci U S A 2004 101 42 15148 15153 10.1073/pnas.0404315101 15471992 PMC523448

[B14] Franke L van Bakel H Fokkens L de Jong ED Egmont-Petersen M Wijmenga C Reconstruction of a functional human gene network, with an application for prioritizing positional candidate genes Am J Hum Genet 2006 78 6 1011 1025 10.1086/504300 16685651 PMC1474084

[B15] Lage K Karlberg EO Storling ZM Olason PI Pedersen AG Rigina O Hinsby AM Tumer Z Pociot F Tommerup N A human phenome-interactome network of protein complexes implicated in genetic disorders Nat Biotechnol 2007 25 3 309 316 10.1038/nbt1295 17344885

[B16] Wu X Jiang R Zhang MQ Li S Network-based global inference of human disease genes Mol Syst Biol 2008 4 189 18463613 10.1038/msb.2008.27 PMC2424293

[B17] Kohler S Bauer S Horn D Robinson PN Walking the interactome for prioritization of candidate disease genes Am J Hum Genet 2008 82 4 949 958 10.1016/j.ajhg.2008.02.013 18371930 PMC2427257

[B18] Vanunu O Magger O Ruppin E Shlomi T Sharan R Associating genes and protein complexes with disease via network propagation PLoS Comput Biol 2010 6 1 e1000641 10.1371/journal.pcbi.1000641 20090828 PMC2797085

[B19] Chen J Aronow BJ Jegga AG Disease candidate gene identification and prioritization using protein interaction networks BMC Bioinformatics 2009 10 73 10.1186/1471-2105-10-73 19245720 PMC2657789

[B20] Chen X Yan GY Liao XP A novel candidate disease genes prioritization method based on module partition and rank fusion OMICS 2010 14 4 337 356 10.1089/omi.2009.0143 20726795

[B21] Sun PG Gao L Han S Prediction of human disease-related gene clusters by clustering analysis Int J Biol Sci 2011 7 1 61 73 21278917 10.7150/ijbs.7.61 PMC3030143

[B22] Lin J Gan CM Zhang X Jones S Sjoblom T Wood LD Parsons DW Papadopoulos N Kinzler KW Vogelstein B A multidimensional analysis of genes mutated in breast and colorectal cancers Genome Res 2007 17 9 1304 1318 10.1101/gr.6431107 17693572 PMC1950899

[B23] Carlson MR Zhang B Fang Z Mischel PS Horvath S Nelson SF Gene connectivity, function, and sequence conservation: predictions from modular yeast co-expression networks BMC Genomics 2006 7 40 10.1186/1471-2164-7-40 16515682 PMC1413526

[B24] Oldham MC Horvath S Geschwind DH Conservation and evolution of gene coexpression networks in human and chimpanzee brains Proc Natl Acad Sci U S A 2006 103 47 17973 17978 10.1073/pnas.0605938103 17101986 PMC1693857

[B25] Navlakha S Kingsford C The power of protein interaction networks for associating genes with diseases Bioinformatics 2010 26 8 1057 1063 10.1093/bioinformatics/btq076 20185403 PMC2853684

[B26] Salwinski L Miller CS Smith AJ Pettit FK Bowie JU Eisenberg D The database of interacting proteins: 2004 update Nucleic Acids Res 2004 32 Database issue D449 451 14681454 10.1093/nar/gkh086 PMC308820

[B27] Alfarano C Andrade CE Anthony K Bahroos N Bajec M Bantoft K Betel D Bobechko B Boutilier K Burgess E The biomolecular interaction network database and related tools 2005 update Nucleic Acids Res 2005 33 Database issue D418 424 15608229 10.1093/nar/gki051 PMC540005

[B28] Aranda B Achuthan P Alam-Faruque Y Armean I Bridge A Derow C Feuermann M Ghanbarian AT Kerrien S Khadake J The IntAct molecular interaction database in 2010 Nucleic Acids Res 2010 38 Database issue D525 531 19850723 10.1093/nar/gkp878 PMC2808934

[B29] Pagel P Kovac S Oesterheld M Brauner B Dunger-Kaltenbach I Frishman G Montrone C Mark P Stumpflen V Mewes HW The MIPS mammalian protein-protein interaction database Bioinformatics 2005 21 6 832 834 10.1093/bioinformatics/bti115 15531608

[B30] Ceol A Chatr Aryamontri A Licata L Peluso D Briganti L Perfetto L Castagnoli L Cesareni G MINT, the molecular interaction database: 2009 update Nucleic Acids Res 2010 38 Database issue D532 539 19897547 10.1093/nar/gkp983 PMC2808973

[B31] Keshava Prasad TS Goel R Kandasamy K Keerthikumar S Kumar S Mathivanan S Telikicherla D Raju R Shafreen B Venugopal A Human Protein Reference Database--2009 update Nucleic Acids Res 2009 37 Database issue D767 772 18988627 10.1093/nar/gkn892 PMC2686490

[B32] Stark C Breitkreutz BJ Reguly T Boucher L Breitkreutz A Tyers M BioGRID: a general repository for interaction datasets Nucleic Acids Res 2006 34 Database issue D535 539 16381927 10.1093/nar/gkj109 PMC1347471

[B33] Matthews L Gopinath G Gillespie M Caudy M Croft D de Bono B Garapati P Hemish J Hermjakob H Jassal B Reactome knowledgebase of human biological pathways and processes Nucleic Acids Res 2009 37 Database issue D619 622 18981052 10.1093/nar/gkn863 PMC2686536

[B34] Cerami EG Gross BE Demir E Rodchenkov I Babur O Anwar N Schultz N Bader GD Sander C Pathway Commons, a web resource for biological pathway data Nucleic Acids Res 2011 39 Database issue D685 690 21071392 10.1093/nar/gkq1039 PMC3013659

[B35] Jensen LJ Kuhn M Stark M Chaffron S Creevey C Muller J Doerks T Julien P Roth A Simonovic M STRING 8--a global view on proteins and their functional interactions in 630 organisms Nucleic Acids Res 2009 37 Database issue D412 416 18940858 10.1093/nar/gkn760 PMC2686466

[B36] von Mering C Huynen M Jaeggi D Schmidt S Bork P Snel B STRING: a database of predicted functional associations between proteins Nucleic Acids Res 2003 31 1 258 261 10.1093/nar/gkg034 12519996 PMC165481

[B37] Hamosh A Scott AF Amberger JS Bocchini CA McKusick VA Online Mendelian Inheritance in Man (OMIM), a knowledgebase of human genes and genetic disorders Nucleic Acids Res 2005 33 Database issue D514 517 15608251 10.1093/nar/gki033 PMC539987

[B38] Barabasi AL Oltvai ZN Network biology: understanding the cell's functional organization Nat Rev Genet 2004 5 2 101 113 10.1038/nrg1272 14735121

[B39] Chung MY Lu YC Cheng NC Soong BW A novel autosomal dominant spinocerebellar ataxia (SCA22) linked to chromosome 1p21-q23 Brain 2003 126 Pt 6 1293 1299 12764052 10.1093/brain/awg130

[B40] Duenas AM Goold R Giunti P Molecular pathogenesis of spinocerebellar ataxias Brain 2006 129 Pt 6 1357 1370 16613893 10.1093/brain/awl081

[B41] Matilla-Duenas A Sanchez I Corral-Juan M Davalos A Alvarez R Latorre P Cellular and molecular pathways triggering neurodegeneration in the spinocerebellar ataxias Cerebellum 2010 9 2 148 166 10.1007/s12311-009-0144-2 19890685

[B42] Schols L Bauer P Schmidt T Schulte T Riess O Autosomal dominant cerebellar ataxias: clinical features, genetics, and pathogenesis Lancet Neurol 2004 3 5 291 304 10.1016/S1474-4422(04)00737-9 15099544

[B43] Lim J Hao T Shaw C Patel AJ Szabo G Rual JF Fisk CJ Li N Smolyar A Hill DE A protein-protein interaction network for human inherited ataxias and disorders of Purkinje cell degeneration Cell 2006 125 4 801 814 10.1016/j.cell.2006.03.032 16713569

[B44] Ikeda Y Dick KA Weatherspoon MR Gincel D Armbrust KR Dalton JC Stevanin G Durr A Zuhlke C Burk K Spectrin mutations cause spinocerebellar ataxia type 5 Nat Genet 2006 38 2 184 190 10.1038/ng1728 16429157

[B45] Lorenzo DN Li MG Mische SE Armbrust KR Ranum LP Hays TS Spectrin mutations that cause spinocerebellar ataxia type 5 impair axonal transport and induce neurodegeneration in Drosophila J Cell Biol 2010 189 1 143 158 10.1083/jcb.200905158 20368622 PMC2854382

[B46] Stankewich MC Gwynn B Ardito T Ji L Kim J Robledo RF Lux SE Peters LL Morrow JS Targeted deletion of betaIII spectrin impairs synaptogenesis and generates ataxic and seizure phenotypes Proc Natl Acad Sci U S A 2010 107 13 6022 6027 10.1073/pnas.1001522107 20231455 PMC2851889

[B47] Clarkson YL Gillespie T Perkins EM Lyndon AR Jackson M Beta-III spectrin mutation L253P associated with spinocerebellar ataxia type 5 interferes with binding to Arp1 and protein trafficking from the Golgi Hum Mol Genet 2010 19 18 3634 3641 10.1093/hmg/ddq279 20603325 PMC2928133

[B48] Sun G Tomita H Shakkottai VG Gargus JJ Genomic organization and promoter analysis of human KCNN3 gene J Hum Genet 2001 46 8 463 470 10.1007/s100380170046 11501944

[B49] Grube S Gerchen MF Adamcio B Pardo LA Martin S Malzahn D Papiol S Begemann M Ribbe K Friedrichs H A CAG repeat polymorphism of KCNN3 predicts SK3 channel function and cognitive performance in schizophrenia EMBO Mol Med 2011 3 6 309 319 10.1002/emmm.201100135 21433290 PMC3377084

[B50] Jin DK Hwang HZ Oh MR Kim JS Lee M Kim S Lim SW Seo MY Kim JH Kim DK CAG repeats of CTG18.1 and KCNN3 in Korean patients with bipolar affective disorder J Affect Disord 2001 66 1 19 24 10.1016/S0165-0327(00)00291-3 11532529

[B51] Aerts S Lambrechts D Maity S Van Loo P Coessens B De Smet F Tranchevent LC De Moor B Marynen P Hassan B Gene prioritization through genomic data fusion Nat Biotechnol 2006 24 5 537 544 10.1038/nbt1203 16680138

[B52] Li Y Patra JC Integration of multiple data sources to prioritize candidate genes using discounted rating system BMC Bioinformatics 2010 11 Suppl 1 S20 10.1186/1471-2105-11-S1-S20 20122192 PMC3009491

[B53] Goh KI Cusick ME Valle D Childs B Vidal M Barabasi AL The human disease network Proc Natl Acad Sci U S A 2007 104 21 8685 8690 10.1073/pnas.0701361104 17502601 PMC1885563

