# Supplementary material for: Prioritizing disease candidate genes by a gene interconnectedness-based approach
Source: BMC Genomics. 2011 Nov 30;12(Suppl 3):S25. doi: 10.1186/1471-2164-12-S3-S25 (PMC3333184; doi:10.1186/1471-2164-12-S3-S25)
Supplement: Additional file 1 — List of related algorithms and tools for prioritizing disease candidate genes [file 1471-2164-12-S3-S25-S1.pdf]

Additional file 1 – Existing approaches and tools for the prediction or prioritization of disease candidate genes

Approach	Description	Data types used[&]	References
Freudenberg and Propping	This study produces clusters of known disease genes based on a measure of phenotype similarity between diseases.	GO, OMIM	[1]
GeneSeeker	It is a web tool that integrates data from mapping, expression and phenotypic database and allows genes meeting user-defined criteria to be retrieved. (http://www.cmbi.ru.nl/GeneSeeker/)	GEXP, ORTH, OMIM	[2-3]
Bortoluzzi et al	This study reconstructs genomic transcriptional profiles of different human tissues and identifies the disease genes by looking for highly expression genes.	GEXP	[4]
POCUS	It prioritizes disease candidate genes by basing on over-representation of functional annotations between loci for the same disease.	GO, PDOM	[5]
DGP	This study develops a computational method that allows the detection of genes likely to be involved in hereditary disease in the human genome based on the sequence properties (i.e. protein length, phylogenetic extent, degree of conservation and paralogy). (http://maine.ebi.ac.uk:8000/services/dgp)	PCON, PHYL, SEQ	[6]
GFINDer	It is a web server for the analysis of genetic disorder related genes by exploiting the information on genetic diseases and their clinical phenotypes present in textual form within the OMIM database. (http://www.medinfopoli.polimi.it/GFINDer)	GO	[7-8]
Huang et al	This study investigates whether human disease genes differ significantly from their orthologs with respect to their overall levels of conservation and their rates of evolutionary change. This study found that disease genes have higher mutation rates over evolutionary time.	GEXP, PHYL, SEQ	[9]

Molecular triangulation	This study uses text mining to capture knowledge of molecular interactions, and then combines shortest path distance on gene interaction network and linkage confidence to prioritize candidate genes.	TXT	[10]
BITOLA	It is an interactive literature-based biomedical discovery support system. The purpose of the system is to help the biomedical researchers make new discoveries by discovering potentially new relations between biomedical concepts. (http://ibmi.mf.uni-lj.si/bitola/)	TXT	[11]
PROSPECTR	It builds a decision tree-based model based on the sequence features for ranking the disease candidate genes (http://www.genetics.med.ed.ac.uk/prospectr/)	PHYL, SEQ	[12]
Tiffin et al	This study uses the eVOC anatomical ontology to integrate text-mining of biochemical literature and data-mining of available human gene expression data.	ONTO (eVOC), OMIM	[13]
D2G	This study develops a sophisticated treatment of the biomedical literature that associates pathological condition with particular GO terms which allow disease candidate genes to be ranked according to the number of these terms they share.	GO, TXT	[14-15]
GeneRank	It is an intuitive modification of PageRank that maintains many of its mathematical properties. It combines gene expression information with a network structure derived from gene annotations (gene ontologies) or expression profile correlations.	GEXP, GO, PPI	[16]
CoGenT++	It is designed to provide a comprehensive, robust, flexible and useful environment guided by research issues in computational genomics. Extensions based on the CoGenT++ environment include disease gene prediction, pattern discovery, automated domain detection, genome annotation and ancestral reconstruction. (http://cgg.ebi.ac.uk/cogentpp.html)	SEQ, PDOM, OMIM	[17]

SUSPECTS	It is a gene prioritization method that ranks genes from a given chromosomal region of interest regarding to a specific disease or a set of candidate genes by matching sequence features, gene expression data, InterPro domains and GO. Each gene is ranked by a weighted score obtained by these four information sources. (http://www.genetics.med.ed.ac.uk/suspects/)	GO, GEXP, PDOM, SEQ, PHYL	[18]
MimMiner	It is a system for text-mining analysis of the human phenome that classifies human disease phenotypes from OMIM and phenotype similarities. It ranks phenotypes by their similarity to a given disease phenotype. (http://www.cmbi.ru.nl/MimMiner/cgi-bin/main.pl)	OMIM	[19]
GFSST	It uses a statistical model to measure the functional similarity of gene based on the GO annotations. (http://gfsst.nci.nih.gov/)	GO	[20]
SNPs3D	SNPs3D is a web resource that provides and integrates as much information as possible on disease-gene relationships at the molecular level. (http://www.snps3d.org/)	GEXP, GO, MP, PATH, PPI, OMIM, TXT	[21]
Oti et al	This study predicts the disease candidate genes by considering interactions with known disease causing genes.	PPI	[22]
Endeavour	It prioritize candidate genes based on similarity between candidate genes and the known disease genes for each data source and then fuses each of these rankings from the separate data source into a single ranking. (http://homes.esat.kuleuven.be/~bioiuser/endeavour/index.php)	GO, GEXP, PATH, PDOM, PPI, SEQ, TFBS, TXT	[23-24]
Prioritizer	It constructs a functional gene network by integrating gene expression, gene ontology, and protein interaction. It prioritizes the disease candidate genes based	GEXP, GO, PATH, PPI	[25]

	on the shortest paths on this integrated gene network. (http://www.prioritizer.nl/)		
TOM	It is a web-based resource for the efficient extraction of candidate genes for hereditary diseases. The service requires the previous knowledge of at least another gene responsible for the disease and the linkage area, or else of two disease associated genetic intervals. (http://www.micrel.deis.unibo.it/~tom/)	GEXP, GO	[26-27]
PhD-SNP	It is based on support vector machines (SVMs) that starting from the protein sequence information can predict whether a new phenotype derived from an nsSNP can be related to a genetic disease in humans. (http://gpcr.biocomp.unibo.it/cgi/predictors/PhD-SNP/PhD-SNP.cgi)	SEQ	[28]
Xu and Li	This study predicts disease genes by building K-nearest neighbors (KNN) algorithm classifier based on the topological properties on protein interaction network.	PPI	[29]
GenTrepid	It combines two methods for automated prediction of disease genes: common pathway scanning (CPS) via protein interaction and common module profiling (CMP) via protein domain composition. (https://www.gentrepid.org/)	PATH, PDOM, PPI	[30]
Chen et al	This study uses an initial gene list for Alzheimer disease to expand it based on protein interaction and proposes a scoring function to identify other Alzheimer disease causing genes.	PPI	[31]
CGI	It prioritizes genes associated with a phenotype by combining gene expression and protein interaction data (CGI).	GEXP, PPI	[32]
CAESAR	It exploits the knowledge of complex traits in literature by using ontologies to semantically map the trait information to gene and protein centric information from several different public data sources. (http://visionlab.bio.unc.edu/Caesar/)	GEXP, GO, ONTH (eVOC, mammalian ontology), PATH, PDOM, PPI, TXT	[33]

Lage et al	This study builds a Bayesian model based on the protein interactions and disease phenotype similarity to predict disease genes.	OMIM, PPI	[34]
aGeneApart	This method creates a set of chromosomal aberration maps that associate cytogenetic bands to biomedical concepts from a variety of controlled vocabularies, including disease, dysmorphology, anatomy, development and Gene Ontology branches. (http://tomcatbackup.esat.kuleuven.be/sanger/)	TXT	[35]
De Bie et al	It develops a multiple kernel learning algorithm to measure the similarity between genes and then uses SVM trained on the combined kernel to prioritize candidate genes.	GEXP, GO, PATH, PDOM, PPI, TXT	[36]
ToppGene	It is a gene prioritization method that combines mouse phenotype data with human gene annotations and literature. It ranks candidate genes based on a similarity score for each annotation of each candidate by comparing to the enriched terms in a given set of training genes. (http://toppgene.cchmc.org/prioritization.jsp)	GEXP, GO, MP, PATH, PDOM, PPI, TFBS, TXT	[37-38]
Liquid association	It applies liquid association algorithm to prioritize disease candidate genes.	GEXP	[39]
Nguyen and Ho	This study proposes a semi-supervised learning approach to integrate protein interaction and various biological data and to predict disease candidate genes.	SEQ, GO, PDOM, PPI	[40]
PGMapper	It is a software tool for automatically matching phenotype to genes. Candidate gene search will be based on the genome region and key words that describe the particular phenotypic features specified by users. (http://www.genediscovery.org/pgmapper/)	GEXP, OMIM	[41]
GeneRanker	It is an online system that allows researchers to obtain a ranked list of genes	OMIM, PPI, TXT	[42]

	potentially related to a specific disease or biological process by combining gene-disease associations with protein-protein interactions extracted from the literature, using computational analysis of the protein network topology to more accurately rank the predicted associations. (http://cbioc.eas.asu.edu/generanker/)		
Mani et al	It uses gene expression data in combination with molecular interaction to identify interactions that exhibit a gain or a loss of expression correlation in a given phenotypic class. It ranked genes according to the enrichment of their direct neighborhood with such interactions.	GEXP, PPI	[43]
PhenoPred	It is based on known gene-disease associations, protein-protein interaction data, protein functional annotation at a molecular level and protein sequence data. Machine learning principles are used to integrate heterogeneous data sources. (http://www.phenopred.org/)	GO, PSTR, PPI, SEQ	[44]
GeneWanderer	It employs a random walk process from known disease genes on the protein interaction network to prioritize disease candidate genes. (http://compbio.charite.de/genewanderer/GeneWanderer)	PPI	[45]
Ala et al	It generates a human-mouse conserved coexpression network (CCN) to prioritize the disease candidate genes.	GEXP	[46]
Oti et al	It uses coexpression data from yeast, worm, fly, mouse, and human and looks for the highly conserved coexpression between candidate genes and the known disease genes.	GEXP	[47]
CIPHER	It scores a candidate gene based on the correlation between the disease phenotypic similarity and gene closeness with other disease genes.	OMIM, PPI	[48]

	(http://bioinfo.au.tsinghua.edu.cn/cipher/)		
PolySearch	It is a web-based text mining system for extracting relationships between human diseases, genes, mutations, drugs and metabolites. (http://wishart.biology.ualberta.ca/polysearch/)	GO, PPI, TXT	[49]
Miozzi et al	It combines gene expression data, functional annotation and know phenotype-gene associations to predict candidate genes.	GEXP, GO, OMIM	[50]
Ozgur et al	This study constructs the gene interaction network through mining literature and uses network topological characteristics to prioritize disease candidate genes.	TXT	[51]
CANDID	It is a prioritization algorithm designed to produce accurate rankings of candidate genes that influence complex human traits. It uses information from publications, protein domain descriptions, cross-species conservation measures, gene expression profiles and protein-protein interactions in its analysis. (https://dsgweb.wustl.edu/hutz/candid.html)	GEXP, PCON, PDOM, PPI, TXT	[52]
CGPrio	It is a resource for the prioritization of candidate cancer genes based on computational classifiers that use different combinations of sequence and functional data including sequence conservation, protein domains and interactions, and regulatory data. (http://bg.upf.edu/cgprio/)	GSTR, PCON, PDOM, PPI	[53]
GeneDistiller	It is a web-based application for prioritizing candidate gene in a linkage interval. Information from various data sources such as gene-phenotype associations, gene expression patterns and protein-protein interactions was integrated into a central database. (http://www.genedistiller.org/)	GEXP, GO, MP, OMIM, PATH, PDOM, PPI, TXT	[54]
Gene	It is a web-based application that selects and prioritizes potential disease-related	TXT	[55]

Prospector	genes by using a highly curated and updated literature database of genetic association studies. (http://www.hugenavigator.net/HuGENavigator/geneProspectorStartPage.do)		
ACGR	It exploits relationships between genes and diseases to lead to the retrieval of documented sets of candidate genes.	GO, ORTH, TXT	[56]
Chen et al	This study applies PageRank, HITS (Hyperlink-induced topic search) algorithms, and K-step Markov method to prioritize disease candidate genes.	PPI	[57]
Karni et al	This study develops an algorithm for predicting disease candidate gene. It relies on the assumption that in the disease state, one or more causal genes are disrupted, leading to the expression changes of downstream.	GEXP, PPI	[58]
PosMed	It prioritizes candidate genes for positional cloning by employing our original database search engine GRASE [59], which uses an inferential process similar to an artificial neural network comprising documental neurons that represent each document contained in databases such as MEDLINE and OMIM. (http://omicspace.riken.jp/PosMed/)	TXT	[60]
FLN	This studies integrates 16 genomics feature to construct an evidence-weighted functional-linkage networks (FLN). The FLN is used to prioritize the disease candidate genes based its evidence-weights.	GEXP, GP, PDOM, PHYL, PPI, ORTH, TXT	[61]
FunSimMat	It is a comprehensive resource of semantic and functional similarity values. It allows ranking disease candidate proteins for OMIM diseases and searching for functional similarity values. (http://www.funsimmat.de/)	GO	[62]
PRINCE	This algorithm is based on formulating constraints on the prioritization function that	PPI	[63]

	relate to its smoothness over the network and usage of prior information.		
RWRH	It applied the random walk with restart algorithm on the heterogeneous network which used the phenotype-gene relationship information from the OMIM to connect the gene network and phenotype network. (http://www3.ntu.edu.sg/home/aspatra/research/Yongjin_BI2010.zip)	OMIM, PPI	[64]
DRS	It proposes a discounted rating system (DRS) to integrate the ranking results from different data sources.	GO, PPI	[65]
MCDGPA	It is a modularized candidate disease gene prioritization algorithm. It includes three steps: module partition, gene prioritization in each disease-associated module, and rank fusion for the global ranking.	PPI	[66]
Sun et al	This study apply clustering algorithms, including Markov cluster algorithm (MCL), molecular complex detection (MCODE), and clique percolation method (CPM), in prioritizing disease candidate genes.	PPI	[67]
MedSim	It is a novel approach for ranking candidate genes for a particular disease based on functional comparisons involving the Gene Ontology. MedSim uses functional annotations of known disease genes for assessing the similarity of diseases as well as the disease relevance of candidate genes (http://www.funsimmat.de)	GO, PPI, ORTH	[68]
Lee & Gonzalez	It proposes a novel, general, and flexible formulation that enables multi-source data integration for gene prioritization that maximizes the complementary nature of different data and knowledge sources in order to make the most use of the information content of aggregate data.	GEXP, GO, PPI	[69]
Jia et al	This study performed an integrative analysis of CNV data, gene association data,	GEXP, PPI, PATH	[70]

	protein-protein interaction, and gene expression data to prioritize candidate genes associated with epilepsy.		
DomainRBF	It is a Bayesian regression approach to prioritize candidate protein domains for human complex disease.	PDOM, PPI	[71]
Nitsch et al	It proposes three strategies scoring disease candidate genes relying on network-based machine learning approaches, such as kernel ridge regression, heat kernel, and Arnoldi kernel approximation.	GEXP, PPI	[72]
PINTA	It is a web resource for the prioritization of candidate genes based on the differential expression of their neighborhood in a genome-wide protein-protein interaction network.	GEXP, PPI	[73]
Xiao et al.	It proposes a novel differential expression pattern (DEP)-based approach integrating numerous disease-specific expression data sets for prioritizing candidate genes.	GEXP	[74]
Lombard et al	The combination of gene annotation information and sequence motif-orientated computational candidate gene prediction methods highlight an added benefit in generating a list of plausible candidate genes.	GO, ONTO, SEQ	[75]
MAXIF	It applies a maximum information flow (MAXIF) method that make use of the phenome-interactome network to calculate an association score for each candidate gene, and then ranks the candidate genes according to their scores.	OMIM, PPI	[76]
RWPCN	The basis of RWPCN is a protein complex network constructed using existing human protein complexes and protein interaction network. The associations between the protein complexes and the query phenotypes in their respective protein complex and phenotype networks are computed to prioritize candidate disease genes for the	OMIM, PPI	[77]

	query disease phenotypes.		

[&]Abbreviations used in data types:

GEXP: gene expression including EST and microarray experiments

GO: Gene Ontology annotation

MP: mouse phenotype information

OMIM: information extracted from OMIM

ONTO: other ontologies

ORTH: including other species data

PATH: pathway

PCOM: protein conservation information

PDOM: protein domain

PHYL: phylogenetic information

PPI protein-protein interaction

PSTR: protein structure

SEQ: sequence properties, such length, composition of amino acids

TFBS: transcriptional factor binding site

TXT: information extracted from biomedical literature

References in Additional file 1

1. Freudenberg J, Propping P: **A similarity-based method for genome-wide prediction of disease-relevant human genes**. *Bioinformatics* 2002, **18 Suppl 2**:S110-115.
2. van Driel MA, Cuelenaere K, Kemmeren PP, Leunissen JA, Brunner HG: **A new web-based data mining tool for the identification of**

candidate genes for human genetic disorders. *Eur J Hum Genet* 2003, **11**(1):57-63.
3. van Driel MA, Cuelenaere K, Kemmeren PP, Leunissen JA, Brunner HG, Vriend G: **GeneSeeker: extraction and integration of human disease-related information from web-based genetic databases**. *Nucleic Acids Res* 2005, **33**(Web Server issue):W758-761.
4. Bortoluzzi S, Romualdi C, Bisognin A, Danieli GA: **Disease genes and intracellular protein networks**. *Physiol Genomics* 2003, **15**(3):223-227.
5. Turner FS, Clutterbuck DR, Semple CA: **POCUS: mining genomic sequence annotation to predict disease genes**. *Genome Biol* 2003, **4**(11):R75.
6. Lopez-Bigas N, Ouzounis CA: **Genome-wide identification of genes likely to be involved in human genetic disease**. *Nucleic Acids Res* 2004, **32**(10):3108-3114.
7. Masseroli M, Martucci D, Pinciroli F: **GFINDer: Genome Function INtegrated Discoverer through dynamic annotation, statistical analysis, and mining**. *Nucleic Acids Res* 2004, **32**(Web Server issue):W293-300.
8. Masseroli M, Galati O, Pinciroli F: **GFINDer: genetic disease and phenotype location statistical analysis and mining of dynamically annotated gene lists**. *Nucleic Acids Res* 2005, **33**(Web Server issue):W717-723.
9. Huang H, Winter EE, Wang H, Weinstock KG, Xing H, Goodstadt L, Stenson PD, Cooper DN, Smith D, Alba MM *et al*: **Evolutionary conservation and selection of human disease gene orthologs in the rat and mouse genomes**. *Genome Biol* 2004, **5**(7):R47.
10. Krauthammer M, Kaufmann CA, Gilliam TC, Rzhetsky A: **Molecular triangulation: bridging linkage and molecular-network information for identifying candidate genes in Alzheimer's disease**. *Proc Natl Acad Sci U S A* 2004, **101**(42):15148-15153.
11. Hristovski D, Peterlin B, Mitchell JA, Humphrey SM: **Using literature-based discovery to identify disease candidate genes**. *Int J Med Inform* 2005, **74**(2-4):289-298.
12. Adie EA, Adams RR, Evans KL, Porteous DJ, Pickard BS: **Speeding disease gene discovery by sequence based candidate prioritization**. *BMC Bioinformatics* 2005, **6**:55.
13. Tiffin N, Kelso JF, Powell AR, Pan H, Bajic VB, Hide WA: **Integration of text- and data-mining using ontologies successfully selects disease gene candidates**. *Nucleic Acids Res* 2005, **33**(5):1544-1552.

14. Perez-Iratxeta C, Bork P, Andrade MA: **Association of genes to genetically inherited diseases using data mining**. *Nat Genet* 2002, **31**(3):316-319.
15. Perez-Iratxeta C, Wjst M, Bork P, Andrade MA: **G2D: a tool for mining genes associated with disease**. *BMC Genet* 2005, **6**:45.
16. Morrison JL, Breitling R, Higham DJ, Gilbert DR: **GeneRank: using search engine technology for the analysis of microarray experiments**. *BMC Bioinformatics* 2005, **6**:233.
17. Goldovsky L, Janssen P, Ahren D, Audit B, Cases I, Darzentas N, Enright AJ, Lopez-Bigas N, Peregrin-Alvarez JM, Smith M *et al*: **CoGenT++: an extensive and extensible data environment for computational genomics**. *Bioinformatics* 2005, **21**(19):3806-3810.
18. Adie EA, Adams RR, Evans KL, Porteous DJ, Pickard BS: **SUSPECTS: enabling fast and effective prioritization of positional candidates**. *Bioinformatics* 2006, **22**(6):773-774.
19. van Driel MA, Bruggeman J, Vriend G, Brunner HG, Leunissen JA: **A text-mining analysis of the human phenome**. *Eur J Hum Genet* 2006, **14**(5):535-542.
20. Zhang P, Zhang J, Sheng H, Russo JJ, Osborne B, Buetow K: **Gene functional similarity search tool (GFSST)**. *BMC Bioinformatics* 2006, **7**:135.
21. Yue P, Melamud E, Moult J: **SNPs3D: candidate gene and SNP selection for association studies**. *BMC Bioinformatics* 2006, **7**:166.
22. Oti M, Snel B, Huynen MA, Brunner HG: **Predicting disease genes using protein-protein interactions**. *J Med Genet* 2006, **43**(8):691-698.
23. Aerts S, Lambrechts D, Maity S, Van Loo P, Coessens B, De Smet F, Tranchevent LC, De Moor B, Marynen P, Hassan B *et al*: **Gene prioritization through genomic data fusion**. *Nat Biotechnol* 2006, **24**(5):537-544.
24. Tranchevent LC, Barriot R, Yu S, Van Vooren S, Van Loo P, Coessens B, De Moor B, Aerts S, Moreau Y: **ENDEAVOUR update: a web resource for gene prioritization in multiple species**. *Nucleic Acids Res* 2008, **36**(Web Server issue):W377-384.
25. Franke L, van Bakel H, Fokkens L, de Jong ED, Egmont-Petersen M, Wijmenga C: **Reconstruction of a functional human gene network, with an application for prioritizing positional candidate genes**. *Am J Hum Genet* 2006, **78**(6):1011-1025.
26. Rossi S, Masotti D, Nardini C, Bonora E, Romeo G, Macii E, Benini L, Volinia S: **TOM: a web-based integrated approach for identification of candidate disease genes**. *Nucleic Acids Res* 2006, **34**(Web Server issue):W285-292.

27. Masotti D, Nardini C, Rossi S, Bonora E, Romeo G, Volinia S, Benini L: **TOM: enhancement and extension of a tool suite for in silico approaches to multigenic hereditary disorders**. *Bioinformatics* 2008, **24**(3):428-429.
28. Capriotti E, Calabrese R, Casadio R: **Predicting the insurgence of human genetic diseases associated to single point protein mutations with support vector machines and evolutionary information**. *Bioinformatics* 2006, **22**(22):2729-2734.
29. Xu J, Li Y: **Discovering disease-genes by topological features in human protein-protein interaction network**. *Bioinformatics* 2006, **22**(22):2800-2805.
30. George RA, Liu JY, Feng LL, Bryson-Richardson RJ, Fatkin D, Wouters MA: **Analysis of protein sequence and interaction data for candidate disease gene prediction**. *Nucleic Acids Res* 2006, **34**(19):e130.
31. Chen JY, Shen C, Sivachenko AY: **Mining Alzheimer disease relevant proteins from integrated protein interactome data**. *Pac Symp Biocomput* 2006:367-378.
32. Ma X, Lee H, Wang L, Sun F: **CGI: a new approach for prioritizing genes by combining gene expression and protein-protein interaction data**. *Bioinformatics* 2007, **23**(2):215-221.
33. Gaulton KJ, Mohlke KL, Vision TJ: **A computational system to select candidate genes for complex human traits**. *Bioinformatics* 2007, **23**(9):1132-1140.
34. Lage K, Karlberg EO, Storling ZM, Olason PI, Pedersen AG, Rigina O, Hinsby AM, Tumer Z, Pociot F, Tommerup N *et al*: **A human phenome-interactome network of protein complexes implicated in genetic disorders**. *Nat Biotechnol* 2007, **25**(3):309-316.
35. Van Vooren S, Thienpont B, Menten B, Speleman F, De Moor B, Vermeesch J, Moreau Y: **Mapping biomedical concepts onto the human genome by mining literature on chromosomal aberrations**. *Nucleic Acids Res* 2007, **35**(8):2533-2543.
36. De Bie T, Tranchevent LC, van Oeffelen LM, Moreau Y: **Kernel-based data fusion for gene prioritization**. *Bioinformatics* 2007, **23**(13):i125-132.
37. Chen J, Xu H, Aronow BJ, Jegga AG: **Improved human disease candidate gene prioritization using mouse phenotype**. *BMC Bioinformatics* 2007, **8**:392.
38. Chen J, Bardes EE, Aronow BJ, Jegga AG: **ToppGene Suite for gene list enrichment analysis and candidate gene prioritization**. *Nucleic*

Acids Res 2009, **37**(Web Server issue):W305-311.
39. Li KC, Palotie A, Yuan S, Bronnikov D, Chen D, Wei X, Choi OW, Saarela J, Peltonen L: **Finding disease candidate genes by liquid association**. *Genome Biol* 2007, **8**(10):R205.
40. Nguyen TP, Ho TB: **A Semi-supervised Learning Approach to Disease Gene Prediction**. In: *Bioinformatics and Biomedicine, 2007 BIBM 2007 IEEE International Conference on: 2-4 Nov. 2007 2007*; 2007: 423-428.
41. Xiong Q, Qiu Y, Gu W: **PGMapper: a web-based tool linking phenotype to genes**. *Bioinformatics* 2008, **24**(7):1011-1013.
42. Gonzalez G, Uribe JC, Armstrong B, McDonough W, Berens ME: **GeneRanker: An Online System for Predicting Gene-Disease Associations for Translational Research**. *Summit on Translat Bioinforma* 2008, **2008**:26-30.
43. Mani KM, Lefebvre C, Wang K, Lim WK, Basso K, Dalla-Favera R, Califano A: **A systems biology approach to prediction of oncogenes and molecular perturbation targets in B-cell lymphomas**. *Mol Syst Biol* 2008, **4**:169.
44. Radivojac P, Peng K, Clark WT, Peters BJ, Mohan A, Boyle SM, Mooney SD: **An integrated approach to inferring gene-disease associations in humans**. *Proteins* 2008, **72**(3):1030-1037.
45. Kohler S, Bauer S, Horn D, Robinson PN: **Walking the interactome for prioritization of candidate disease genes**. *Am J Hum Genet* 2008, **82**(4):949-958.
46. Ala U, Piro RM, Grassi E, Damasco C, Silengo L, Oti M, Provero P, Di Cunto F: **Prediction of human disease genes by human-mouse conserved coexpression analysis**. *PLoS Comput Biol* 2008, **4**(3):e1000043.
47. Oti M, van Reeuwijk J, Huynen MA, Brunner HG: **Conserved co-expression for candidate disease gene prioritization**. *BMC Bioinformatics* 2008, **9**:208.
48. Wu X, Jiang R, Zhang MQ, Li S: **Network-based global inference of human disease genes**. *Mol Syst Biol* 2008, **4**:189.
49. Cheng D, Knox C, Young N, Stothard P, Damaraju S, Wishart DS: **PolySearch: a web-based text mining system for extracting relationships between human diseases, genes, mutations, drugs and metabolites**. *Nucleic Acids Res* 2008, **36**(Web Server issue):W399-405.
50. Miozzi L, Piro RM, Rosa F, Ala U, Silengo L, Di Cunto F, Provero P: **Functional annotation and identification of candidate disease genes**

by computational analysis of normal tissue gene expression data. *PLoS One* 2008, **3**(6):e2439.
51. Ozgur A, Vu T, Erkan G, Radev DR: **Identifying gene-disease associations using centrality on a literature mined gene-interaction network**. *Bioinformatics* 2008, **24**(13):i277-285.
52. Hutz JE, Kraja AT, McLeod HL, Province MA: **CANDID: a flexible method for prioritizing candidate genes for complex human traits**. *Genet Epidemiol* 2008, **32**(8):779-790.
53. Furney SJ, Calvo B, Larranaga P, Lozano JA, Lopez-Bigas N: **Prioritization of candidate cancer genes--an aid to oncogenomic studies**. *Nucleic Acids Res* 2008, **36**(18):e115.
54. Seelow D, Schwarz JM, Schuelke M: **GeneDistiller--distilling candidate genes from linkage intervals**. *PLoS One* 2008, **3**(12):e3874.
55. Yu W, Wulf A, Liu T, Khoury MJ, Gwinn M: **Gene Prospector: an evidence gateway for evaluating potential susceptibility genes and interacting risk factors for human diseases**. *BMC Bioinformatics* 2008, **9**:528.
56. Yilmaz S, Jonveaux P, Bicep C, Pierron L, Smail-Tabbone M, Devignes MD: **Gene-disease relationship discovery based on model-driven data integration and database view definition**. *Bioinformatics* 2009, **25**(2):230-236.
57. Chen J, Aronow BJ, Jegga AG: **Disease candidate gene identification and prioritization using protein interaction networks**. *BMC Bioinformatics* 2009, **10**:73.
58. Karni S, Soreq H, Sharan R: **A network-based method for predicting disease-causing genes**. *J Comput Biol* 2009, **16**(2):181-189.
59. Kobayashi N, Toyoda T: **Statistical search on the Semantic Web**. *Bioinformatics* 2008, **24**(7):1002-1010.
60. Yoshida Y, Makita Y, Heida N, Asano S, Matsushima A, Ishii M, Mochizuki Y, Masuya H, Wakana S, Kobayashi N *et al*: **PosMed (Positional Medline): prioritizing genes with an artificial neural network comprising medical documents to accelerate positional cloning**. *Nucleic Acids Res* 2009, **37**(Web Server issue):W147-152.
61. Linghu B, Snitkin ES, Hu Z, Xia Y, Delisi C: **Genome-wide prioritization of disease genes and identification of disease-disease associations from an integrated human functional linkage network**. *Genome Biol* 2009, **10**(9):R91.
62. Schlicker A, Albrecht M: **FunSimMat update: new features for exploring functional similarity**. *Nucleic Acids Res* 2010, **38**(Database issue):D244-248.

63. Vanunu O, Magger O, Ruppin E, Shlomi T, Sharan R: **Associating genes and protein complexes with disease via network propagation**. *PLoS Comput Biol* 2010, **6**(1):e1000641.
64. Li Y, Patra JC: **Genome-wide inferring gene-phenotype relationship by walking on the heterogeneous network**. *Bioinformatics* 2010, **26**(9):1219-1224.
65. Li Y, Patra JC: **Integration of multiple data sources to prioritize candidate genes using discounted rating system**. *BMC Bioinformatics* 2010, **11 Suppl 1**:S20.
66. Chen X, Yan GY, Liao XP: **A novel candidate disease genes prioritization method based on module partition and rank fusion**. *OMICS* 2010, **14**(4):337-356.
67. Sun PG, Gao L, Han S: **Prediction of human disease-related gene clusters by clustering analysis**. *Int J Biol Sci* 2011, **7**(1):61-73.
68. Schlicker A, Lengauer T, Albrecht M: **Improving disease gene prioritization using the semantic similarity of Gene Ontology terms**. *Bioinformatics* 2010, **26**(18):i561-567.
69. Lee JH, Gonzalez GH: **Towards integrative gene prioritization in Alzheimer's disease**. *Pac Symp Biocomput* 2011:4-13.
70. Jia P, Ewers JM, Zhao Z: **Prioritization of epilepsy associated candidate genes by convergent analysis**. *PLoS One* 2011, **6**(2):e17162.
71. Zhang W, Chen Y, Sun F, Jiang R: **DomainRBF: a Bayesian regression approach to the prioritization of candidate domains for complex diseases**. *BMC Syst Biol* 2011, **5**:55.
72. Nitsch D, Goncalves JP, Ojeda F, de Moor B, Moreau Y: **Candidate gene prioritization by network analysis of differential expression using machine learning approaches**. *BMC Bioinformatics* 2010, **11**:460.
73. Nitsch D, Tranchevent LC, Goncalves JP, Vogt JK, Madeira SC, Moreau Y: **PINTA: a web server for network-based gene prioritization from expression data**. *Nucleic Acids Res* 2011.
74. Xiao Y, Xu C, Ping Y, Guan J, Fan H, Li Y, Li X: **Differential expression pattern-based prioritization of candidate genes through integrating disease-specific expression data**. *Genomics* 2011.
75. Lombard Z, Park C, Makova KD, Ramsay M: **A Computational Approach to Candidate Gene Prioritization for X-Linked Mental Retardation using Annotation-based Binary Filtering and Motif-Based Linear Discriminatory Analysis**. *Biol Direct* 2011, **6**(1):30.

76. Chen Y, Jiang T, Jiang R: **Uncover disease genes by maximizing information flow in the phenome-interactome network**. *Bioinformatics* 2011, **27**(13):i167-i176.
77. Yang P, Li X, Wu M, Kwoh CK, Ng SK: **Inferring Gene-Phenotype Associations via Global Protein Complex Network Propagation**. *PLoS One* 2011, **6**(7):e21502.
