# Supplementary figures and images for: Prioritizing disease candidate genes by a gene interconnectedness-based approach
Source: BMC Genomics. 2011 Nov 30;12(Suppl 3):S25. doi: 10.1186/1471-2164-12-S3-S25 (PMC3333184; doi:10.1186/1471-2164-12-S3-S25)

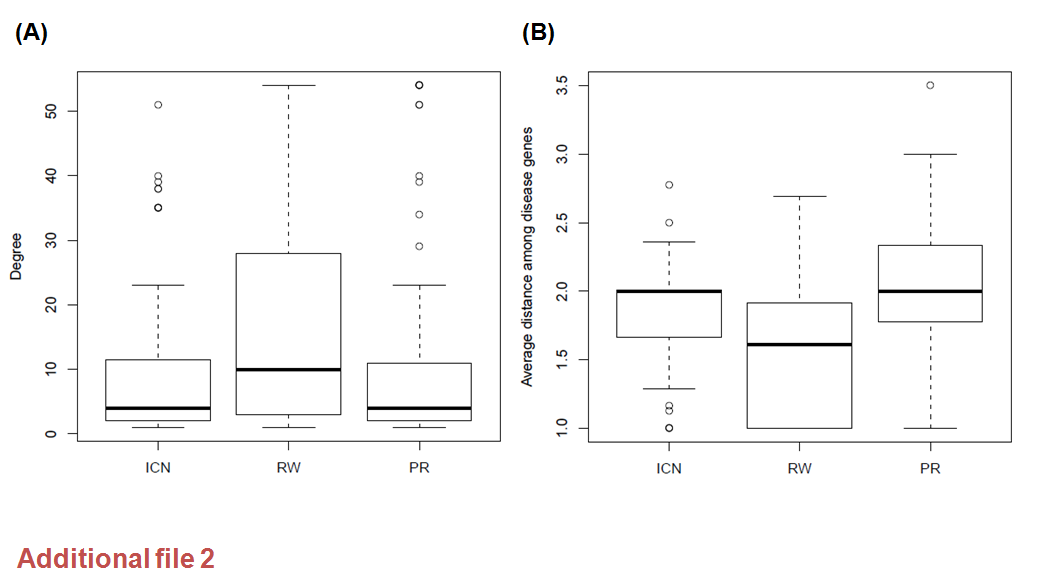

Supplement: Additional file 2 — Analysis of network topological properties on disease causing genes The topological properties of disease genes in unique cases which were successfully ranked the known disease genes as top 1 candidate by a specific method in FAN (Figure 3B) were compared in degree (A) and average shortest-path distance between other disease-associated genes which are in the same disease family(B). [file 1471-2164-12-S3-S25-S2.tif]
